# Chinese herbal medicine *Shenzhuo* Formula treatment in patients with macroalbuminuria secondary to diabetic kidney disease: study protocol for a randomized controlled trial

**DOI:** 10.1186/s13063-018-2573-z

**Published:** 2018-03-27

**Authors:** Xue-min Zhao, Ying Zhang, Xin-hui He, Hong-dong Chen, Zhu-feng Wang, Jing Guo, Xin-miao Wang, Ze-zheng Gao, Ji-ping Wang, Wei Liu, Lin-hua Zhao, Xiao-lin Tong

**Affiliations:** 1 Guang’anmen Hospital, China Academy of Chinese Medical Sciences, Beijing, 100053 China; 2 Center for Evidence-based Chinese Medicine, Beijing University of Chinese Medicine, Beijing, 100029 China; 3 South area of Guang’anmen Hospital, China Academy of Chinese Medical Sciences, Beijing, 100000 China; 4 Beijing University of Chinese Medicine, Beijing, 100029 China; 5 Dongzhimen Hospital, Beijing University of Chinese Medicine, Beijing, 100700 China; 6 Hepingli Hospital, Beijing, 100013 China; 7 Zouping County Hospital of TCM, Binzhou, 256200 China; 8 Yongzhou Central Hospital, Yongzhou, 425000 China

**Keywords:** Traditional Chinese medicine, Diabetic kidney disease, 24-hour urinary protein, Randomized controlled trials, Macroalbuminuria

## Abstract

**Background:**

Diabetic kidney disease (DKD) is a serious complication associated with diabetes mellitus and can cause end-stage renal disease (ESRD). Traditional Chinese medicine (TCM) is widely used in China to treat DKD, and in particular microalbuminuria and macroalbuminuria. This study will address the efficacy and safety of *Shenzhuo* Formula (SZF), a frequently prescribed TCM, in DKD patients with macroalbuminuria.

**Methods/design:**

This study is a 24-week, randomized, multi-center, double-blinded, double-dummy, controlled, clinical trial that will include 120 DKD patients aged 18 to 80 years old with a 24-h urinary protein (24-h UP) level of between 0.5 g and 3 g and serum creatinine (SCr) ≤ 133 μmol/L (1.5 mg/dL) and compare SZF to irbesartan. The 24-h UP change from baseline to week 24 will represent the primary endpoint with secondary endpoints including SCr, estimated glomerular filtration rate (eGFR), TCM symptoms, urinary albumin excretion rate (UAER), etc. Safety assessments will also be evaluated.

**Discussion:**

This study will provide initial evidence regarding the efficacy and safety of SZF relative to irbesartan in the treatment of DKD patients with macroalbuminuria.

**Trial registration:**

Chinese Clinical Trial Registry, ID: ChiCTR-ICR-15006311. Registered on 15 April 2015.

**Electronic supplementary material:**

The online version of this article (10.1186/s13063-018-2573-z) contains supplementary material, which is available to authorized users.

## Background

Diabetic kidney disease (DKD) is one of the most common complications of diabetes and also the leading cause of chronic kidney disease (CKD) and end-stage renal disease (ESRD) in many countries, including the US. In China, DKD is, after glomerulonephropathy, the second leading cause of ESRD [1]. In parallel with type-2 diabetes, the incidence of DKD continues to increase [2]. The costs of care for patients with DKD are extremely high, especially when it proceeds to ESRD, placing an increasing burden on both the finance and health care systems.

Preventing progression to ESRD in patients with DKD requires, especially in the overt proteinuria period, prompt identification and management [3]. Randomized trials have demonstrated that renin-angiotensin-aldosterone system (RAAS) blockers can reduce albuminuria and the progression to renal failure in DKD [4]. Side effects of RAAS blocker agents, including angioedema, persistent cough, temporary increases in serum creatinine (SCr), compromise patients’ long-term compliance [5, 6]. Thus, exploring additional interventions that could achieve equivalent or superior impact on renal function in DKD patients while avoiding adverse effects remains worthy of exploration.

Traditional Chinese medicine (TCM) is a medical system founded on syndrome-pattern differentiation, and defining the main TCM syndrome type of DKD is essential to the treatment processes [7]. Based on long-term clinical observations, we have classified the most common signs and symptoms of DKD as deficiency of *qi*, and blood stasis; other study results support this classification [8, 9]. The effects of SZF replenish *qi* and promote blood circulation. According to this main syndrome type, reports from Chinese medical practitioners, the herbs of *Shenzhuo* Formula (SZF) show promise for the treatment of DKD.

In recent years, large-scale observational studies have provided further evidence of the efficacy and safety of TCM in glycemic control of diabetes mellitus (DM) and renoprotection against CKD [10–12]. A 5-year retrospective study has provided initial evidence that SZF may slow DKD progression by improving the estimated glomerular filtration rate (eGFR) [13]. A second retrospective analysis of 2 years of follow-up has provided additional evidence that modified SZF can reduce 24-hour urinary protein (24-h UP) excretion in DKD patients and may be able to improve eGFR and blood lipid levels [14]. There is also clinical evidence from a randomized controlled trial (RCT) of the power of TCM in treatment of type-2 diabetic kidney disease with macroalbuminuria [15].

In another retrospective study, SZF was used to treat 63 DKD patients with microalbuminuria, and the results showed that after intervention for 3 months and 6 months, microalbuminuria decreased in 92.1% and 90.5% of patients, respectively. In that study, levels of glycosylated (HbA1_C_), systolic blood pressure (SBP), and total cholesterol (TC) showed significant reductions across the same time frame [16]. Another retrospective study provided further support for the action of SZF in slowing the progression of DKD, and, to a certain extent, controlling blood levels of glucose, lipids, and blood pressure (BP) [1].

Despite these encouraging results, the relative effects of TCM versus RAAS blockers in DKD remain, however, very limited, and thus require further exploration. This study is designed to examine the efficacy and safety of SZF and RAAS in the treatment of DKD patients with macroalbuminuria.

## Methods/design

### Study design

#### Ethics, consent, and permissions

The Ethics Committee of Guang’anmen Hospital has approved (Approval No. 2015EC038) the protocol (version identifier: SZF (20150304)) which is registered on the Chinese Clinical Trial Registry (http://www.chictr.org.cn/showproj.aspx?proj=10862) under number ChiCTR-ICR-15006311. The study will be conducted in accordance with the principles of the Declaration of Helsinki (2013 version). The study design will comply with the principles set out in the Good Clinical Practice (GCP) guidelines according to the theory that guides the appropriate use of TCM in clinical application. The trial will be completed according to the Standard Protocol Items: Recommendations for Intervention Trials (SPIRIT) guidelines [17] (see Additional file 1). Informed consent will be obtained from all participants prior to entry into the trial including knowledge of the purpose of the trial and the possible risks and benefits. All visits will be documented in Case Report Forms (CRFs).

#### Study design and settings

**Fig. 1:**
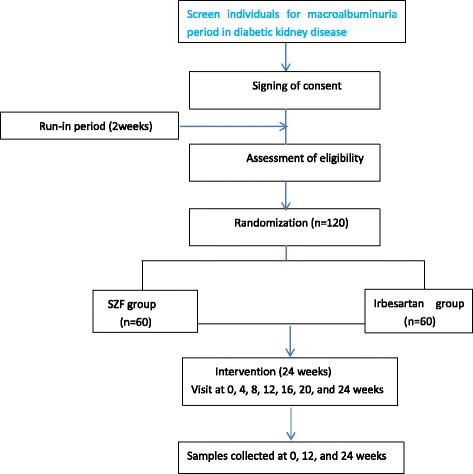
Flow diagram of progress through the study

This study is to be a 26-week, randomized, multi-center, double-blinded, controlled clinical trial, with a 2-week washout period and 24-week treatment period comparing SZF to irbesartan. The trial flow, including measurements every 4 weeks and collection of biological samples at 0, 12, and 24 weeks is illustrated in Fig. 1. Nine hospitals in China have agreed to participate in the study: Guang’anmen Hospital of China Academy of TCM, Beijing; Dongzhimen Hospital of Beijing University of TCM, Beijing; the Jishuitan Hospital of TCM Affiliated with Peking University, Beijing; Baoding Hospital of TCM, Baoding; Shijiazhuang Hospital of TCM, Shijiazhuang; Tongzhou Hospital of TCM, Beijing; Shantou Hospital of TCM, Shantou; Yongzhou Central Hospital, Yongzhou; and Zouping County Hospital of TCM, Binzhou.

### Participants

Participants will require a diagnosis of DM, a diagnosis of DKD, and a diagnosis of deficiency of *qi* with blood stasis.

#### Diagnostic criteria

Diagnostic criteria for DM are based on the criteria set by the World Health Organization in 1999 [18] and the American Diabetes Association (ADA) criteria in 2013 [19]. Diagnostic criteria for DKD are based on Clinical Practice Guidelines and Clinical Practice Recommendations for Diabetes and Chronic Kidney Disease established by the American Kidney Foundation in 2007 and 2012 [20, 21], as shown in Table 2.

#### TCM syndrome pattern differentiation

The Chinese syndrome pattern differentiation type indicating deficiency of *qi* with blood stasis will be based on guidelines delineated in the Clinical Research of New Investigational Drugs in Traditional Chinese Medicine [22], and Guidelines for the Prevention and Treatment of Diabetes in Traditional Chinese Medicine. The diagnostic standards are as follows:

1. Primary signs and symptoms include fatigue, listlessness, weakness, and dry mouth and throat
2. Secondary signs and symptoms include soreness of the lower back and knees; spontaneous perspiration and sensation of heat in the palms and soles; and dark purplish lips

Participants will be diagnosed with deficiency of *qi* and blood stasis syndrome if they have two or more of the primary signs or symptoms, at least two of the secondary signs or symptoms, and examination of the tongue and pulse indicates blood stasis. Investigators at each site will administer a symptom assessment survey to every participant (Table 1). This will ensure standardized scoring across sites.

**Table 1:** Traditional Chinese medicine (TCM) symptom assessment survey

Physical and mental fatigue	□ 0: None □ 2: Easy fatigability □ 4: Reluctance to perform the activities of daily living □ 6: Weakness of limbs
Dry mouth and throat	□ 0: None □ 2: Mild □ 4: Decreased saliva production □ 6: Severe thirst with constant need to drink fluids
Weakness and soreness of the lower back and knees	□ 0: None □ 2: Occasional □ 4: Need to alter body position for relief □ 6: Persistent pain, need to take pain medication
*Qi* deficiency and listlessness	□ 0: None □ 2: Shortness of breath after mild activity □ 4: Shortness of breath after moderate activity □ 6: Inability to speak or catch breath even when still
Spontaneous perspiration	□ 0: None □ 2: Skin slightly wet when not moving □ 4: Skin wet when not moving □ 6: Profuse perspiration when moving
Night sweat	□ 0: None □ 2: Occasional, head only □ 4: Often on the, chest and back □ 6: Always, all over the body
Dysphoria in chest, palms, and soles	□ 0: None □ 2: Occasionally be perturbed □ 4: Often, dryness heat within the body □ 6: Always, need to grasp something cold
Dark purplish lips	□ 0: None □ 2: Pale and dark lips □ 4: Dark lips □ 6: Dark purple lips
Edema	□ 0: None □ 2: Palpebral edema □ 4: Palpebral or edema of the legs □ 6: Hyposarca

After being screened for inclusion and exclusion criteria (Table 2), eligible patients will be entered into the trial.

**Table 2:** Eligibility criteria

Inclusion criteria	Exclusion criteria
• Diabetic kidney disease (DKD) diagnostic criteria:	•Type 1 diabetes
(a) Type-2 diabetes over 10 years with microalbuminuria	• Taking potassium-sparing diuretics
(b) Diabetic retinopathy with microalbuminuria	• Severe anemia, Hb < 60 g/L
(c) Macroalbuminuria: 24-h urinary protein (24-h UP) 0.5–3 g	• Albumin < 35 g/L
•Chinese syndrome differentiation of *qi* deficiency and with blood stasis	• Severe cardiovascular and cerebrovascular diseases
• Age between 18 and 80 years	• Other primary or secondary renal diseases (e.g., IgA nephropathy, membranous nephropathy, or lupus nephritis)
• Willingness to provide signed informed consent	• Pregnant or lactating women, women intending to become pregnant and women not using appropriate contraceptive methods
• HbA1_C_ ≤ 8.0%	• Allergy to components of agents used in the study
• Serum creatinine < 133 μmol/L (1.5 mg/dL)	• Severe mental disorder
• Controlled hypertension (blood pressure ≤ 140/90 mmHg)	• Recent participation in other clinical trials recently
	• Other unstable elements such as malignancy, or migration
	• Liver dysfunction (ALT or AST 2.5 times higher than normal levels)

### Interventions

Eligible participants will be randomized to either the SZF or the irbesartan group. Study medication will include either two packets of the SZF (herb granule, 12 g per packet) and two grains of simulated irbesartan (capsule, 75 mg per grain) in the TCM group or the SZF simulation agents and two irbesartan capsules in the western medicine group twice daily for 24 weeks.

SZF consists of six natural herbs: *Dahuang* (*Rheum palmatum* L.), *Shuizhi* (*Hirudo* powder), *Huangqi* (*Astragalus membranaceus* (Fisch.) Bge.)), *Danshen* (*Radix salviae miltiorrhizae*), *Yinyanghuo* (*Herba epimedii*), and *Yimucao* (*Leenurus heterophyllus*). Both SZF formula and SZF simulation agents are prescription granules which will be provided by Jiangyin Tian Jiang Pharmaceutical Co., Ltd. (Jiangyin, China) and distributed by Guang’anmen Hospital of Chinese Academy of traditional Chinese medicine. Irbesartan capsules and irbesartan capsule simulation agents will be provided by the Hongsheng Pharmaceutical Company (Hangzhou, China) and Bailing Pharmaceutical Company (Guiyang, China), respectively.

The manufacturing process will be quality controlled to ensure that the packets will have consistent purity, microbial content, weight, and other physical characteristics. Irbesartan capsules and irbesartan capsule simulation agents will be also rendered consistent with respect to physical characteristics. The manufacturer will provide certificates of quality for the manufacturing of drugs in the SZF group and the irbesartan group. The main components in SZF are validated and quantified by thin-layer chromatography and high-performance liquid chromatography.

Both SZF and SZF simulation agents are manufactured as fine brown granules with the same smell and packaged in packets identical in appearance. When dissolved in warm water, the taste, smell, and appearance of the SZF and SZF simulation agents are identical. Both the irbesartan capsule and irbesartan simulation agents are in the same appearance, both blue and white capsules. Investigators and participants will be unaware of the drug allocation.

The Investigational Medicinal Product (IMP) will only be dispensed to participants who have provided written informed consent into our trial and who have successfully completed all screening inclusion/exclusion criteria. Only qualified personnel in each site may dispense drugs to participants and they will guarantee that the medicine is kept sealed in a cool, dry, and secure locked place.

Drug accountability will be completed in each site on all returned IMP and on unfinished/empty IMP packages at every visit. Relevant counts and return dates will be recorded in the IMP log.

#### Concomitant treatments

Based on the guidelines issued by the American Diabetes Association (ADA) in 2007, all participants will receive standard treatments, such as antihypertensive drugs, to maintain a blood pressure of ≤ 140/90 mmHg. The recommended drugs are non-dihydropyridine calcium-channel blockers or, alternatively, beta-blockers. The standard treatments include blood glucose control therapy as well: the fasting blood glucose (FBG) of all participants will be controlled to ≤ 7.8 mmol/L and 2-h plasma glucose (2-h PG) will be controlled to ≤ 11.1 mmol/L throughout the trial. Moreover, the participants will, unless it is necessary, not change the routine drugs which are used for their chronic condition.

Participants will be advised to follow a healthy low-sodium, low-fat diet, and take regular exercises. Investigators will be instructed to record the details of any additional/transformational drug or therapy, such as the name, dosage, and duration of administration, in the CRF.

#### Prohibited concomitant medications

During the trial period, no other drugs that could affect urinary protein or renal function will be permitted. These include TCMs that aim to replenish *qi* or promote blood circulation, or drugs such as potassium-sparing diuretics and other kinds of angiotensin-converting enzyme inhibitors or RAABs.

### Study visits and assessment

**Fig. 2:**
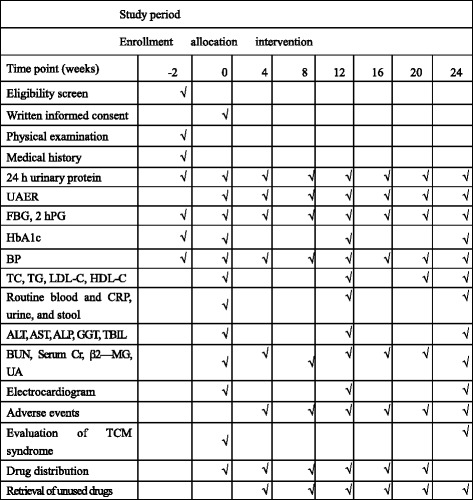
Schedule of enrollment, allocation, visits, and assessments

The study duration will be 26 weeks, comprising a 2-week run-in period and 24 weeks of intervention. After treatment commenced, visits will take place every 4 weeks during the intervention period. An overview of specific measurements and time points of data collection can be found in Fig. 2.

### Outcome measurements

#### Primary outcome

The primary endpoint is the change in 24-h UP from baseline to week 24. Biochemical measurements of 24-h UP will be performed in the laboratory of each hospital every 4 weeks.

#### Secondary outcomes

Secondary outcomes will be as follows:

1. Change in SCr and eGFR (2009 CKD-EPI creatinine equation [23]) from baseline to week 24
2. Change in urinary albumin excretion rate (UAER) from baseline to week 24
3. Improvement in TCM symptoms (evaluation by TCM Symptom Score Scale) from baseline to week 24
4. Change in FBG and 2-h postprandial plasma glucose (2-h PG) from baseline to week 24
5. Change in blood lipids, including the total cholesterol (TC), triglyceride (TG), high-density lipoprotein cholesterol (HDL-C), and low-density lipoprotein cholesterol (LDL-C), from baseline to week 12 and week 24
6. Change in HbA1c from baseline to week 12 and week 24
7. Change in blood pressure (BP) from baseline to week 24

#### Safety assessment

The following safety assessment will be performed at baseline, week 12 and week 24:

1. The results of routine blood, urine, and stool tests
2. Electrocardiogram (ECG)
3. Liver function, including alanine aminotransferase (ALT), aspartate aminotransferase (AST), alkaline phosphatase (ALP), gamma-glutamyl transferase (GGT), and serum total bilirubin (TBIL)
4. Renal function, including blood urea nitrogen (BUN), SCr, uric acid (UA), and β_2_-microglobulin
5. Adverse events (AEs), such as signs and symptoms and other ailments, will be documented at every visit. Every AE will be described as a mild, moderate, or severe AE, and association with the intervention drugs will be assessed. Severe AEs will be reported to the main investigator and the Ethics Committee within 24 h. All the adverse events will be recorded, monitored, and treated until resolved, so other required inspection items such as computed tomography (CT) and ultrasonography (US) will be needed when the discomfort amounts to an AE. We will make between one and six occasional visits during the study to ensure that each participating center complies with the study protocol

#### Other assessments

Demographic information will be collected at baseline.

#### Biological sample collection

In order to study and analyze the therapeutic mechanism underlying SZF in DKD, fecal, blood, and urine samples will be collected at 0, 12, and 24 weeks.

The blood and urine samples in each hospital will be collected on site, and will be tested and analyzed on site. The plasma of each blood sample will be separated by centrifugation (3000 prn, 15 min, 4 °C), and the serum of each blood sample will be extracted after being allowed to stand for some time. In order to ensure specimen quality, all samples are to be packed in freezing tubes and stored in liquid nitrogen for at least 3 min prior to being transferred to a − 80 °C refrigerator at each site. If the detection outcomes in different hospitals have relevant differences, we plan to use cold-chain transportation for central detection.

#### Sample size estimation

Sample size has been calculated based on the primary endpoint and average change of 24-h UP from baseline to week 24. Previous studies suggest a mean difference (MD) of 0.2 g between groups, and the same standard deviation (SD) of 0.3 g for each group. We expect that a total of 100 subjects will be needed to uncover any difference between groups with at least a power of 90%, controlling the type I error rate at 0.05. Considering a dropout rate of 20%, the target sample size will be 120.

#### Randomization and allocation

Participants will be randomly assigned to either the SZF or the irbesartan group at a 1:1 ratio. The stratified block randomization will be adopted, regarding center as strata factor. SAS software will be used to automatically generate the random sequence. The Institute of Basic Research in Clinical Medicine of the China Academy of Chinese Medical Sciences will be responsible for the drug blinding and randomization concealment. Everyone concerned in the process of randomization code generation and drug blinding will work independent of the data collection, evaluation, and analysis. The drug administrator at each participating medical site will enroll patients sequentially based on screening order. To ensure concealment, the block sizes will not be disclosed. Both participants and investigators will be kept blinded to the allocation until the research is completed. The statistician will not be involved in random sequence generation.

Twenty-four-hour emergency code break and medical information will be provided by The Institute of Basic Research in Clinical Medicine of the China Academy of Chinese Medical Sciences. Each randomized subject also will be provided with telephone numbers of investigators on duty for any emergency contact.

#### Statistical analysis

Statistical SPSS 16.0 software will be used for data analysis. The primary analysis is to be conducted in the intention-to-treat (ITT) population. All statistical tests are two-sided tests, and *P* < 0.05 will be considered statistically significant. Continuous data will be presented with mean ± SD, and *n* (percentage) for categorical data. Considering that the baseline 24-h UP is essential to the progression of urinary protein, analysis of covariance (ANCOVA) using baseline 24-h UP as covariates is to be performed to analyze the 24-h UP change from baseline to week 24. Interactions between site and group will also be tested.

#### Data collection and management

Investigators who will participate in the study are all qualified physicians, including chief physicians, visiting staffs and residents of the Endocrine Department from all sites. Investigators will be trained in standard operating procedures (SOPs) for trial execution, scrutinization of TCM symptoms, biological sample collection, and handling. According to the original observation records, investigators at all sites will finish the CRFs completely and correctly in a timely manner.

The investigators who come from the center of Guang’anmen Hospital will visit each site regularly to confirm the quality of data collection, and resolve any issue encountered in sites. All documents will be properly classified and preserved under confidential conditions and archived.

#### Participant retention and withdrawal

Participants may withdraw from the research project for any reason at any time, and the reason will be recorded in the CRFs. The investigator will tell patients that they have the right to withdraw from the trial and they will be provided with standardized treatment to ensure their safety under the following circumstances: (1) rapidly decreasing eGFR to less than 30; or ALT or AST twice higher than the normal limits; or SCr beyond the normal range after 4 weeks of treatment; (2) development of severe complications or exacerbation of an existing health condition; (3) poor compliance by participants, such as actual drug usage is less than 80% or more than 120% of the prescribed dose; and (4) use of proscribed drugs during the study.

Participants in this trial will be provided with drugs and scheduled physical examination for free. Some necessary examinations or treatments will also be given for any adverse event.

## Discussion

Traditional Chinese Medicine is a valuable medical treatment that still serves the Chinese population today. Syndrome differentiation and treatment make up the quintessence of TCM. Today, TCM is prescribed by almost all physicians of TCM in China as a basic or complementary therapy for kidney disease. SZF has shown a promising effect in the treatment of DKD [1] and is usually prescribed to treat DKD patients with the common syndrome pattern of deficiency in *qi* and with blood stasis. We therefore undertake this trial to examine the efficacy and safety of SZF in relation to irbesartan for the treatment of DKD.

The quality of the RCTs conducted on TCM therapy is generally poor [24]. Issues include poor study design and methodology, and a lack of training of the investigators and participants. To guarantee the quality of this study and verify the conclusions, the experimental design and study implementation have been enforced under strict quality control. The Consolidated Standards of Reporting Trials (CONSORT) Extension for Chinese Herbal Medicine Formulas [25] has been used to plan the study design. A training session for each site will be held to explain the study protocol, explain how the TCM syndrome pattern differentiation should be performed, and illuminate the SOPs. An independent laboratory at each participating site has been established to process the biochemical measurements.

The findings of this trial may provide an alternative treatment for DKD patients with macroalbuminuria. It may also provide evidence and a scientific explanation for the use of SZF to treat the proteinuria associated with clinical DKD.

## Trial status

The trial has been initiated in August 2014 and is scheduled to be completed in June 2018. Currently, 50 patients have been recruited.

## Additional file

Additional file 1: SPIRIT Checklist. (DOC 128 kb)

## Abbreviations

2-h PG: 2-h postprandial plasma glucose;
AEs: Adverse events
ALP: Alkaline phosphatase
ALT: Alanine aminotransferase
AST: Aspartate aminotransferase
BUN: Blood urea nitrogen
DM: Diabetes mellitus
ECG: Electrocardiogram
eGFR: Estimated glomerular filtration rate
ESRD: End-stage renal disease
FBG: Fasting blood glucose
GGT: gamma-glutamyl transferase
HbA1c: Glycosylated hemoglobin
HDL-C: High-density lipoprotein cholesterol
LDL-C: Low-density lipoprotein cholesterol
SCr: Serum creatinine
SOPs: Standard operating procedures
TBIL: Total bilirubin
TC: Total cholesterol
TCM: Traditional Chinese medicine
TG: Triglycerides
UA: Uric acid
UAER: Urinary albumin excretion rate
